# Advanced renal cell carcinoma (RCC) management: an expert panel recommendation from the Latin American Cooperative Oncology Group (LACOG) and the Latin American Renal Cancer Group (LARCG)

**DOI:** 10.1007/s00432-020-03236-4

**Published:** 2020-05-14

**Authors:** Andrey Soares, Fernando Sabino Marques Monteiro, Fernando Cotait Maluf, Diogo Assed Bastos, Denis Leonardo Jardim, André Deeke Sasse, Adriano Gonçalves e Silva, André P. Fay, Diogo Augusto Rodrigues da Rosa, Evanius Wierman, Fabio Kater, Fabio A. Schutz, Fernando Nunes Galvão de Oliveira, Igor Alexandre Protzner Morbeck, José Augusto Rinck, Karine Martins da Trindade, Manuel Caitano Maia, Vinicius Carrera Souza, Deusdedit Cortez Vieira da Silva Neto, Felipe de Almeida e Paula, Fernando Korkes, Gustavo Franco Carvalhal, Lucas Nogueira, Roni de Carvalho Fernandes, Rodolfo Borges dos Reis, Wagner Eduardo Matheus, Wilson Francisco Schreiner Busato, Walter Henriques da Costa, Stênio de Cássio Zequi

**Affiliations:** 1 Hospital Israelita Albert Einstein, Av. Albert Einstein, 627-Morumbi, São Paulo, SP 05652-900 Brazil; 2 Centro Paulista de Oncologia/Oncoclínicas, Av. Brigadeiro Faria Lima, 4300-Vila Olímpia, São Paulo, SP 01452-000 Brazil; 3 Hospital Santa Lúcia, SHLS 716 Conjunto C, Brasília, DF 70390-700 Brazil; 4 Hospital Universitário de Brasília, SGAN 605, Brasília, DF 70840-901 Brazil; 5 Beneficência Portuguesa de São Paulo, R. Martiniano de Carvalho, 965-Bela Vista, São Paulo, SP 01323-001 Brazil; 6 Hospital Sírio-Libanês, R. Dona Adma Jafet, 91-Bela Vista, São Paulo, SP 01308-050 Brazil; 7 Grupo SOnHE, Av. Dr. Heitor Penteado, 1780-Taquaral, Campinas, SP 13075-460 Brazil; 8 Instituto do Câncer e Transplante de Curitiba (ICTR), R. Myltho Anselmo da Silva, 870-Mercês, Curitiba, PR 80510-130 Brazil; 9 Escola de Medicina e Hospital São Lucas da Pontifícia Universidade Católica do Rio Grande do Sul, Av. Ipiranga, 6690-Prédio 60-Partenon, Porto Alegre, RS 90610-000 Brazil; 10 Grupo Oncoclínicas, R. Tobias da Silva, 126-Moinhos do Vento, Porto Alegre, RS 90570-020 Brazil; 11 Grupo Oncoclínicas, Praia de Botafogo, 300-Botafogo, Rio de Janeiro, RJ 22250-905 Brazil; 12 Instituto de Oncologia do Paraná, R. Mateus Leme, 2631/B-Centro Cívico, Curitiba, PR 80520-174 Brazil; 13 CLION-GRUPO CAM, R. Altino Serbeto de Barros, 119-Itaigara, Salvador, BA 41810-570 Brazil; 14 Hospital Sírio-Libanês, SGAS 613, Centro Médico L2-Asa Sul, Brasília, DF 70200-730 Brazil; 15 AC Camargo Cancer Center, R. Professor Antônio Prudente, 211-Liberdade, São Paulo, SP 01509-010 Brazil; 16 Hospital São Carlos/Oncocentro, Av. Pontes Vieira, 2531-Dionísio Torres, Fortaleza, CE 60135-237 Brazil; 17 Santa Casa de Misericórdia de Fortaleza, R. Barão do Rio Branco, s/n-Centro, Fortaleza, CE 60025-060 Brazil; 18 Centro de Oncologia do Paraná, Rodovia BR-277, 1437-Ecoville, Curitiba, PR 82305-100 Brazil; 19 Oncologia D’Or., Av. São Rafael, 2152, 6 Andar, Hospital São Rafael, São Marcos, Salvador, BA 41253-190 Brazil; 20 Hospital Central da Santa Casa de Misericórdia de São Paulo, R. Dr. Cesário Mota Júnior, 112-Vila Buarque, São Paulo, SP 01221-020 Brazil; 21 Hospital Regional do Câncer de Presidente Prudente, Av. Coronel José Soares Marcondes, 2380-Vila Euclides, Presidente Prudente, SP 19013-050 Brazil; 22 ABC Medical School, Av. Príncipe de Gales, 821-Príncipe de Gales, Santo André, SP 09060-650 Brazil; 23 Hospital das Clínicas da Universidade Federal de Minas Gerais, Av. Prof. Alfredo Balena, 110-Santa Efigência, Belo Horizonte, BH 30130-100 Brazil; 24 Faculdade de Ciências Médicas da Santa Casa de São Paulo, R. Dr. Cesário Mota Jr., 61-Vila Buarque, São Paulo, SP 01221-020 Brazil; 25 Faculdade de Medicina de Ribeirão Preto-Universidade de São Paulo, Av. Bandeirantes, 3900-Monte Alegre, Ribeirão Preto, SP 14049-900 Brazil; 26 Faculdade de Ciências Médicas da Universidade Estadual de Campinas, R. Tessália Vieira de Camargo, 126-Cidade Universitária Zeferino Vaz, Campinas, SP 13083-887 Brazil; 27 UNIVALI-Universidade do Vale do Itajaí, R. Uruguai, 458-Centro, Itajaí, SC 88302-901 Brazil; 28 National Institute for Science and Technology in Oncogenomics and Therapeutic Innovation, AC Camargo Cancer Center, R. Professor Antônio Prudente, 211-Liberdade, São Paulo, SP 01509-010 Brazil

**Keywords:** Renal cell carcinoma, Recommendations, Treatment, Cytoreductive nephrectomy, Immunotherapy

## Abstract

**Purpose:**

The outcome of RCC has improved considerably in the last few years, and the treatment options have increased. LACOG-GU and LARCG held a consensus meeting to develop guidelines to support the clinical decisions of physicians and other health professionals involved in the care of RCC patients.

**Methods:**

Eighty questions addressing relevant advanced RCC treatments were previously formulated by a panel of experts. The voting panel comprised 26 specialists from the LACOG-GU/LARCG. Consensus was determined as 75% agreement. For questions with less than 75% agreement, a new discussion was held, and consensus was determined by the majority of votes after the second voting session.

**Results:**

The recommendations were based on the highest level of scientific evidence or by the opinion of the RCC experts when no relevant research data were available.

**Conclusion:**

This manuscript provides guidance for advanced RCC treatment according to the LACOG-GU/LARCG expert recommendations.

**Electronic supplementary material:**

The online version of this article (10.1007/s00432-020-03236-4) contains supplementary material, which is available to authorized users.

## Introduction

Medical research has led to significant improvements in the treatment of renal cell carcinoma (RCC) and has transformed the outlook for patients with renal cancer. Advances in targeted therapy and immunotherapy (Choueiri and Motzer 2017) represent a watershed in the history of the treatment of this disease. Furthermore, the detection of small renal masses (SRMs) has increased in recent decades, indicating the increased use of imaging tests [computed tomography (CT) and magnetic resonance imaging (MRI)], which are generally performed for unrelated indications (Znaor et al. 2015).

More recently, targeted therapies, such as the introduction of immunotherapy, were implemented to increase the chances of improvement in RCC patients. This medical advance, combined with conventional therapies, resulted in a reduction in the mortality rate in RCC patients in developed countries (Siegel et al. 2017). In Brazil, according to the International Agency for Research on Cancer (IARC), an agency of the World Health Organization (WHO), the estimated age-standardized incidence rate for kidney cancer (both sexes, all ages) was 4.3 per 100.000 people in 2018 (WHO 2018).

Considering the new knowledge about RCC biology, two collaborative groups aimed to review and detail the available clinical data on the treatment and management of renal cancer and to provide treatment recommendations. The Latin American Cooperative Oncology Group–Genitourinary section (LACOG-GU) and Latin American Renal Cancer Group (LARCG) gathered specialists in the field and discussed several questions, such as the role of cytoreductive nephrectomy, metastasectomy and the treatment of advanced disease of different histologic subtypes. The results and recommendations are described in this manuscript. These recommendations are targeted mostly to clinical oncologists and urologists for decision making in everyday renal cancer treatment scenarios and should not replace multidisciplinary discussions or the physician’s impressions of specific cases.

## Methods

The expert meeting occurred in August 2018 in São Paulo, Brazil. Many relevant clinical questions regarding the management of small renal masses and localized disease and the treatment of advanced disease were previously formulated by a committee dedicated to these topics. In this manuscript, we summarized the recommendations established for advanced RCC.

The expert panel was formed by 26 specialists from the LACOG-GU and LARCG. The panel comprised 11 urologic surgeons and 15 clinical oncologists specializing in urologic oncology.

The panel of experts gathered in a conference room where the questions were read and projected. The panel was asked to vote for one of up to nine alternatives presented below the question, one of which was always “abstain” between the possible alternatives. All “abstain” answers were excluded from analysis to calculate the percentage of recommendation. Each panelist had a keypad for voting, and voting was conducted electronically. Each question was read in its entirety, and after the reading was finished, the panel had 10 s to vote.

If at least 75% of the panel agreed with an answer, a consensus was established. If concordance was less than 75% in the first round of voting, the question was put on hold, and a later debate and new round of voting was held at the end of the session to attempt a new consensus. After the new discussion, a recommendation was determined by majority agreement. The recommendations described in this manuscript are the results of the first or second rounds of voting on each question. However, on August 16, 2019, the Brazilian Health Regulatory Agency (ANVISA), under Resolution-RE No. 2282, approved the immunotherapy pembrolizumab (anti-PD-1) in combination with axitinib (tyrosine kinase inhibitor (TKI)) for the treatment of the first-line for patients with advanced or metastatic renal cell carcinoma (ANVISA 2019). Faced with this novelty, the experts decided to carry out a new online vote to include this treatment in the relevant questions. This new voting followed the same procedures as the previous one. In total, 73 questions were raised to address all relevant points about advanced RCC and 56% of the questions reached consensus in the first vote. For reviewing the percentage of votes for each question, see the supplementary material.

All answers described in this manuscript are justified by their level of evidence, according to a predefined classification, adapted from the Oxford Centre for Evidence-based Medicine–Levels of Evidence (Tables 1 and 2) (CEBM 2019).

**Table 1:** Levels of evidence. [Adapted from CEBM (2019)]

Level 1	Systematic reviews (SRs) of randomized controlled trials (RCTs) or RCTs with narrow confidence intervals
Level 2	SRs of cohort studies, individual cohort studies, low-quality RCTs or ecological studies
Level 3	SRs of case–control studies or a single case–control study
Level 4	Case series or low-quality case–control studies or cohort studies
Level 5	Expert opinion

**Table 2:** Levels of recommendation. [Adapted from CEBM (2019)]

Recommendation A	Consistent level 1 studies
Recommendation B	Consistent level 2 or 3 studies or extrapolation from level 1 studies
Recommendation C	Level 4 studies or extrapolation from level 2 or 3 studies
Recommendation D	Level 5 evidence

## Results

### Cytoreductive nephrectomy: still the standard of care?

Until recently, cytoreductive nephrectomy (CN) had been the standard of care for patients with metastatic RCC (mRCC) and a primary renal tumor in place at presentation (Flanigan et al. 2001; Mickisch et al. 2001). Randomized trials leading to this recommendation were conducted during the cytokine era and demonstrated that CN resulted in a significant improvement in overall survival (OS). However, the understanding of renal cancer biology and the improved results with targeted therapies and immunotherapy have recently created a new dilemma for whether the physician should initiate treatment with systemic therapy or start with cytoreductive surgery (Mejean et al. 2018) followed by systemic therapy.

Therefore, the decision between systemic therapy and surgery should be made after discussion by multidisciplinary teams and the evaluation of several factors related to the patient and the disease (Mejean et al. 2018). Cytoreductive nephrectomy can be considered before the initiation of systemic therapy when clinically feasible in selected patients: low-risk patients and selected intermediate-risk patients with good performance status and low metastatic burden, especially patients who are candidates for metastasectomy or surveillance after surgery. Patients presenting with IMDC/MSKCC poor-risk disease, symptomatic metastasis, and hepatic and/or bone and/or brain and/or multi-visceral metastasis should not receive cytoreductive nephrectomy as the initial treatment modality (recommendation level B) (Flanigan et al. 2001; Mickisch et al. 2001). However, cytoreductive nephrectomy can be considered for intermediate-risk patients who do not require immediate systemic therapy (Mejean et al. 2018).

In all other patients not included in the group described above, systemic therapy with a vascular endothelial growth factor (VEGF) receptor inhibitor or immunotherapy should be initiated after multidisciplinary discussion, without a loss of oncologic benefit in terms of OS (recommendation level A) (Mejean et al. 2018). It is important to note that, for patients with good responses to systemic therapy, cytoreductive nephrectomy can be considered later. The procedure is considered safe in this setting according to the literature, although a consensus for its indication is not clear (Bex et al. 2019). With the advent of modern immunotherapy as a standard of care for first-line therapy, the applicability of the results of the Carmena trial is controversial. Whether we should proceed to immunotherapy regardless of surgery or should include surgery in circumstances of good responses to immunotherapy remains an unanswered clinical question. We hope that future studies such as the PROBE trial (Harshman et al. 2019), the PROSPER trial (Harshman 2017) and the TARIBO trial (Procopio 2015) will help us answer this question.

In symptomatic primary tumors (with bleeding and/or urinary obstruction and/or refractory pain and/or hypertensive crisis), palliative nephrectomy should be considered regardless of risk criteria, either before or after the initiation of systemic therapy (recommendation level A) (Mejean et al. 2018). Additionally, the removal of the primary tumor (cytoreductive or debulking nephrectomy) in the context of metastatic disease is not indicated for all patients (recommendation level A) (Bex et al. 2019; Flanigan et al. 2001; Mejean et al. 2018; Mickisch et al. 2001).

### Metastasectomy: for whom?

The resection of metastatic disease, also called metastasectomy, could be recommended after multidisciplinary discussion. Metastasectomy is a treatment option that can be recommended for patients with late relapses after prior nephrectomy for localized RCC, patients with oligometastatic disease at presentation who are also being considered for CN or long-term responders to systemic therapy (Kavolius et al. 1998; Ouzaid et al. 2019; Sun et al. 2018).

Metastasectomy may improve OS. However, the evidence that indicates this fact comes from retrospective studies, systematic reviews, and meta-analyses. There are no prospective randomized data to indicate the benefits of this treatment in patients with mRCC (Apollonio et al. 2019). In a retrospective study of the literature, the OS rate was highest for patients who underwent metastasectomy. Regardless of whether the surgery achieved complete or partial resection of the lesions, the survival rate of patients undergoing any surgical procedure was better than that of patients not undergoing surgery (Kavolius et al. 1998). More recently, a systematic review of 8 published cohort studies concluded that the average OS increased from 36.5 to 142 months in 958 mRCC patients undergoing complete surgical metastasectomy compared to 8.4–27 months in 1309 mRCC patients who underwent incomplete surgical metastasectomy. Furthermore, compared to incomplete metastasectomy, complete surgery improved mortality (*p* < 0.001) (Zaid et al. 2017).

The strongest predictors of survival were good performance status (Eastern Cooperative Oncology Group (ECOG) performance status of 0 or 1), the absence of prior systemic therapy, a disease-free interval from nephrectomy to the detection of metastases of greater than 1 year and a single metastatic site. Those predictors revealed that patients with a lower risk score according to the International Metastatic Database Consortium (IMDC) (Heng et al. 2009) were the ones that derived better survival.

Therefore, the panelists recommend consideration of metastasectomy for low-risk and selected intermediate-risk (IMDC) patients with a disease-free interval from nephrectomy to the detection of metastases of greater than 1 year, a single metastatic site, lymph node metastasis, or bone metastasis—especially a single bone metastasis (recommendation level C) (Kavolius et al. 1998).

The specialist group does not recommend metastasectomy routinely in intermediate-risk and high-risk patients (recommendation level C) (Kavolius et al. 1998). For patients with multiple metastatic sites, brain metastasis or a disease-free interval from nephrectomy to the detection of metastases less than 1 year, there is no formal recommendation for metastasectomy, and each case should be discussed individually (recommendation level D) (Kavolius et al. 1998). It is important to remember that treating SNC metastasis is key before initiating systemic treatment.

### First-line treatment for advanced RCC: what to choose?

A watershed in the treatment of metastatic renal cell cancer was achieved with the discovery of angiogenic molecular pathways driving tumorigenesis and the identification of molecules to block these pathways (Escudier et al. 2007; Motzer et al. 2007). A second turning point in the treatment of RCC was the application of immunotherapy with checkpoint (PD-L1) inhibitors, which improved OS in patients previously treated with angiogenesis inhibitors and demonstrated immune-mediated mechanisms of tumor control (Motzer et al. 2015a, b). However, it is consensus among the panelists that the expression of PD-L1 is not important for defining the first-line systemic treatment of metastatic clear cell carcinoma (recommendation level A) (Motzer et al. 2015a, b, 2018a; Pignon et al. 2019). Clinical studies that sought to predict the success of RCC treatment solely dependent on PD-L1 expression obtained controversial data or there was no association between expression of the biomarker of interest with the treatment used (Motzer et al. 2015a, b, 2018a; Pignon et al. 2019). Predicting treatment benefit based on immune biomarkers may be a reality in the future, but certainly within a complex context such as advanced RCC, a set of different biomarkers (a composite of biomarkers) will be required. It is unlikely that a single molecule, such as PD-L1, will be enough. Besides, these composite biomarkers will need to be prospectively validated in the context of therapeutic clinical trials.

For patients with intermediate-risk or poor-risk disease according to the IMDC score (Escudier et al. 2007), independent of PD-L1 status, the panelists suggest treatment with a combination of nivolumab plus ipilimumab (an anti-PD-1 plus anti-CTLA-4 agents) or pembrolizumab plus axitinib, according to local access to therapies (recommendation level A) (Motzer et al. 2018a, b; Powles et al. 2019a, b). The CheckMate 214 study found that compared to sunitinib (50 mg) orally once daily for 4 weeks (over a 6-week cycle), immunotherapy combined with anti-PD1/CTLA4 agents resulted in significant improvements in the primary objectives of OS and objective response rate (ORR) and numerically progression-free survival in intermediate/poor-risk patients score in a primary analysis of 25.2 months and a follow-up analysis of 32.4 months (Motzer et al. 2018a, b, 2019a, b). Furthermore, a latest update of 42-month follow-up reaffirmed the benefits of the combination of nivolumab plus ipilimumab in relation to sunitinib in intermediate/poor risk patients. In this population, the ORR (42% and 26%; *p* < 0.0001) and the OS (47.0 vs 26.6 months; *p* = 0.0001) were also better in the nivolumab plus ipilimumab group than in the sunitinib group. The complete response rate was also significantly different between the groups (10% in nivo ipi arm versus 1% in sunitinib arm group; *p* = 0.01) (Tannir et al. 2020). The first analysis in the subgroup of favorable-risk patients suggested improved OS and progression-free survival (PFS) rates with sunitinib as compared to ipi/nivo (Motzer et al. 2018a, b), but after extended follow-up of 32.4 and 42 months, this difference was no longer statistically significant (Motzer et al. 2019a, b; Tannir et al. 2020). On the other hand, the combination of pembrolizumab plus axitinib significantly improved OS, PFS and response rate as first-line treatment compared with sunitinib in patients with all IMDC risk groups, regardless of PD-L1 expression, according to the results of study KEYNOTE-426 (Powles et al. 2019a, b).

The rapid advances in checkpoint inhibitor immunotherapy, combined checkpoint inhibitor immunotherapy, and the association between immunotherapy and VEGF-TKI therapy have led to the evaluation of these combinations in the context of first-line treatment (Atkins et al. 2018; Motzer et al. 2018a, b). Recent data suggest that the combination of these modalities might be the future of renal cell cancer treatment and renders high-dose interleukin-2 (Fyfe et al. 1995) treatment of no additional benefit in the current renal oncology landscape (recommendation level A) (Atkins et al. 2018; Motzer et al. 2015a, b. 2018a, b).

Still based on scientific evidence from the KEYNOTE-426 study, the panelists recommend that patients with the good-risk disease, independent of PD-L1 status, should also be treated with the combination of immunotherapy plus TKI (pembrolizumab plus axitinib) (recommendation level A) (Powles et al. 2019a, b).

Cabozantinib, another VEGF-TKI, recently approved in Brazil, might be the first choice for treatment in a minority of intermediate- or poor-risk patients with predominantly bone metastatic disease and patients with contraindications to immunotherapy or without access to immunotherapy (recommendation level B) (Choueiri et al. 2015, 2016, 2018; McKay et al. 2014). Cabozantinib was shown to be especially effective in bone disease, which is associated with poor prognosis (Choueiri et al. 2018), in clinical and pre-clinical studies (Choueiri et al. 2014; Graham et al. 2014), warranting its consideration in this context. Cabozantinib was compared with sunitinib in first-line therapy in the prospective phase II trial named CABOSUN (Choueiri et al. 2015, 2018). OS was increased with cabozantinib, although the difference was not statistically significant (hazard ratio [HR], 0.80; 95% CI, 0.53–1.21). However, in the CABOSUN trial, PFS, the primary endpoint, was significantly prolonged with cabozantinib (median, 8.6 vs 5.3 months; HR, 0.48; 95% CI, 0.31–0.74) (Choueiri et al. 2015, 2018).

The antiangiogenic drug of choice between pazopanib and sunitinib for most panelists is pazopanib, due to toxicity profile (recommendation level A) (Motzer et al. 2013a, b). Although sunitinib was noninferior to pazopanib with respect to PFS, the safety and quality-of-life profiles favor pazopanib (Motzer et al. 2013a, b). Importantly, the recently presented data demonstrating the superiority of avelumab plus axitinib over sunitinib in terms of PFS (Choueiri et al. 2019) were not available at the time of the consensus meeting and during the internet voting. Avelumab plus axitinib was recently approved in first line clear cell RCC (ccRCC) in Brazil, and is also available for patients, although it remains to be demonstrated a statistically significant improvement in OS (Motzer et al. 2019a, b).

For patients with no possibility of immunotherapy or cabozantinib treatment, even intermediate or poor-risk patients, the panelists recognize that either pazopanib or sunitinib should be a treatment option for first-line therapy.

### Second-line treatment in metastatic disease: how to proceed?

For patients who progress after initial immunotherapy (PD-L1 + CTLA4 dual checkpoint blockade), the panelists suggest VEGF-TKI therapy (recommendation level C) (Choueiri et al. 2015; Choueiri et al. 2016; Choueiri et al. 2018; Escudier et al. 2007; Escudier et al. 2014; Motzer et al. 2007,2013a, b; Rini et al. 2011). Options include cabozantinib, axitinib, sunitinib, pazopanib, and combination therapy with lenvatinib plus everolimus.

Cabozantinib is a TKI that targets the VEGF receptor and inhibits expression of the MET and AXL genes, which are associated with resistance to VEGF molecular pathway inhibitors and poor prognosis in RCC patients (Choueiri et al. 2014). Cabozantinib was compared with everolimus as second-line therapy in the METEOR trial (Choueiri et al. 2015, 2016). In this trial, OS was significantly prolonged with cabozantinib (median, 21.4 vs 17.1 months; HR, 0.70; 95% CI, 0.58–0.85). Observational studies suggest that cabozantinib is active irrespective of prior exposure to PD-1/PD-L1 treatments or IMDC risk groups (Auvray et al. 2019). Recent data confirm that TKI therapy maintains its benefit after IO therapy (Powles et al. 2018). Therefore, most panelists believe in cabozantinib superiority as a second-line treatment and recommend this medication after progression on first-line immunotherapy with nivolumab plus ipilimumab or pembrolizumab plus axitinib (recommendation level B) (Auvray et al. 2019; Powles et al. 2018). Cabozantinib was also recommended after progression on VEGF-TKI therapy (recommendation level A) (Auvray et al. 2019; Choueiri et al. 2015, 2016; Powles et al. 2018). The recommendation of cabozantinib after VEGF-TKI therapy is due to the optimal control of the disease, with progressive disease as the best response in only 9% of the patients, which makes this treatment an option for patients with rapidly progressive disease (recommendation level A) (Choueiri et al. 2015, 2016).

Axitinib is an inhibitor of VEGF receptors 1, 2, and 3 (Motzer et al. 2013a, b; Rini et al. 2011). Axitinib was compared with sorafenib as second-line treatment in the AXIS trial and resulted in a significant improvement in PFS (median, 8 vs 6 months; HR, 0.66; 95% CI, 0.55–0.78). A significant increase in the ORR with axitinib (23 vs 12%) was observed in the trial, but no significant difference in OS was detected (Motzer et al. 2013a, b; Rini et al. 2011). Axitinib was also compared with sorafenib as first-line treatment in a phase III trial but did not improve PFS or OS and was associated with higher toxicity rates (Hutson et al. 2013). Also, in a single-institution experience, axitinib demonstrated activity after PD-1/PD-L1 checkpoint inhibitor therapy and prior VEGF-TKI therapy (Auvray et al. 2019). The panelists recommend axitinib as second-line treatment after progression on therapy with the VEGF-TKIs sunitinib or pazopanib if cabozantinib is not accessible or is contraindicated (recommendation level B) (Motzer et al. 2013a, b; Ornstein et al. 2018; Rini et al. 2009) and as an option after progression on IO/IO combo (recommendation level C) (Motzer et al. 2013a, b). The panelists agreed that axitinib may also be considered after cabozantinib and PD-1/PD-L1 checkpoint inhibitor therapy (recommendation level C) (Motzer et al. 2013a, b; Ornstein et al. 2018; Rini et al. 2009).

In developing and underdeveloped countries, access to immunotherapy and new VEGF-TKI therapies are extremely difficult. Therefore, sequential inhibition of the VEGF pathway is an alternative for the management of mRCC cases. Clinical data suggest that tumors do not appear to be cross-resistant to sequential therapy with different VEGF-TKI pathway inhibitors (Di Lorenzo et al. 2009; Rautiola et al. 2014; Rini et al. 2009), and thus patients can be exposed to sequential therapy with different VEGF-TKI pathway inhibitors and demonstrate activity. For example, sorafenib was used in patients who had progressed on sunitinib, with objective response rates of 10% and a median time to progression of 16 weeks (Di Lorenzo et al. 2009). Another example is the use of pazopanib after sunitinib therapy in a single-center experience, in which 19% of the patients (95% CI, 12–26%) achieved a partial response with pazopanib after sunitinib therapy (Rautiola et al. 2014). Therefore, the panelists recommend that a minority of patients could be treated by sequential therapy with different VEGF-TKIs, particularly due to access or cost reasons (recommendation level D) (Di Lorenzo et al. 2009; Rautiola et al. 2014; Rini et al. 2009).

Additionally, mTOR pathway inhibition showed positive results in early treatment trials for mRCC. Everolimus is an orally administered mTOR inhibitor and has activity in patients with advanced RCC (Motzer et al. 2008, 2010). In the RECORD-1 phase III trial, everolimus was compared with placebo plus best supportive care in patients progressing on VEGF-TKI. The median PFS for everolimus was 4.9 months, versus 1.9 months for placebo (HR, 0.33; *p* < 0.001) as assessed by independent central review. These results established the efficacy of everolimus in patients with mRCC after progression on sunitinib and/or sorafenib (Motzer et al. 2010, 2014).

However, two recent randomized trials showed that everolimus is significantly less effective than either cabozantinib or nivolumab in previously treated patients (Choueiri et al. 2015; Motzer et al. 2015a, b). Therefore, the panelists do not recommend everolimus after progression on VEGF-TKIs or checkpoint immunotherapy (recommendation level D) (Choueiri et al. 2015; Motzer et al. 2010, 2008, 2014, 2015a, b). However, everolimus could still be considered in areas where second-line VEGFR-TKIs are not available or after two previous lines of VEGF-TKI and/or IOs.

Lenvatinib is a multitarget TKI first tested in thyroid cancer (Cabanillas et al. 2015). Its activity in renal cancer was tested in a phase II trial comparing lenvatinib alone, lenvatinib in combination with everolimus, and everolimus alone in metastatic or locally advanced mRCC patients who progressed after VEGF-TKI therapy (Motzer et al. 2015a, b). The primary endpoint of the study was PFS, which was significantly prolonged with combination therapy (lenvatinib plus everolimus) compared to therapy with everolimus alone (14.6 vs 5.5 months; HR 0.40; *p* = 0.0005) but showed only a trend to be prolonged compared to that with lenvatinib alone (7.4 months; HR 0.66; *p* = 0.12). OS was higher with the combination therapy (18.5 months) than with the therapy of either everolimus (16.5 months) or lenvatinib (17.8 months) alone, even though none of these differences were statistically significant. Interestingly, the ORR was also increased in the combination group (43%) compared with the everolimus (6%; *p *< 0.0001) or lenvatinib alone groups (27%; *p* = 0.10). Nonetheless, adverse events affected fewer patients treated with everolimus alone (50%) than patients treated with lenvatinib alone (79%) or lenvatinib plus everolimus (71%). In May 2016, the combination of lenvatinib plus everolimus was approved by the US Food and Drug Administration (FDA) as an option therapy for patients progressing on VEGF-TKI therapy (Eisai Inc. 2016). The panelists recommend lenvatinib plus everolimus in a minority of patients after progression on VEGF-TKI therapy (recommendation level B) (Eisai Inc. 2016). The recommendation after VEGF-TKI therapy is due to optimal control of the disease, with progressive disease as the best response in only 4% of the patients, rendering this combination an option for patients with rapidly progressive disease (recommendation level B) (Eisai Inc. 2016; Motzer et al. 2015a, b).

### Third-line treatment in metastatic disease: how to proceed?

The combination of lenvatinib plus everolimus was recommended as the third-line treatment for patients who progressed after immunotherapy/immunotherapy combination or immunotherapy plus axitinib as first-line treatment and a TKI as second-line treatment (recommendation level D) (Motzer et al. 2015a, b). This same combination was also recommended for patients who progressed after treatment with cabozantinib (first-line) and nivolumab (second-line) (recommendation level D) (Motzer et al. 2015a, b). However, there is no strong scientific evidence to support lenvatinib plus everolimus as a third line of treatment for mRCC since this combination was tested as a second-line option in the pivotal trial mentioned previously (Motzer et al. 2015a, b).

For patients who have disease progression after one or two VEGF inhibitors therapy the panelists recommend nivolumab over everolimus treatment (recommendation level A) (Motzer et al. 2015a, b). The CheckMate025 phase III study the treatment with nivolumab 3 mg/kg every 2 weeks was superior in patients with mRCC who received previous anti-angiogenic therapy. In this study, the objective response rate was greater (25% vs 5%) and the OS was 25.0 vs 19.6 months favoring nivolumab. Furthermore, the third and further treatment line-related adverse events were lower in the nivolumab group (Motzer et al. 2015a, b).

A phase III trial comparing tivozanib (a potent and selective inhibitor of VEGF 1, 2 and 3) with sorafenib in the third-line setting (TIVO-3 study) was recently reported (Rini et al. 2019) and demonstrated the superiority of tivozanib in terms of PFS, with a median PFS of 5.6 months versus 3.9 months for tivozanib and sorafenib, respectively (HR, 0.73; 95% CI, 0.56–0.94; *p* = 0.0165). Additionally, the 1-year and 2-year PFS rates were 28% versus 11% and 18% versus 5%, respectively, both favoring tivozanib. The ORR was 18% for tivozanib and 8% for sorafenib (*p* = 0.02). Although tivozanib has not yet been approved in Brazil and, therefore, not discussed in the consensus meeting, we believe that tivozanib may have a role in the third-line setting, including for patients progressing after TKI therapy and immunotherapy (Rini et al. 2019).

### Non-clear cell RCC (nccRCC) histology: what to do?

Renal cell cancer is classified according to the cell of origin, morphology, growth pattern, histochemical characteristics and molecular profile (Patard et al. 2005; Storkel and van den Berg 1995). The different histological subtypes of RCC include clear cell, papillary (chromophilic) type I and type II, chromophobe, oncocytic, collecting duct (Bellini's duct), unclassified RCC, translocation carcinoma and medullary carcinoma (Moch et al. 2016; Vera-Badillo et al. 2015). There is also sarcomatoid RCC (sRCC), which is not considered a separate histologic subtype, because sarcomatoid features can be seen in any histologic subtype of RCC (Kavolius et al. 1998).

Data from the literature show that patients with both nccRCC and ccRCC may respond to VEGF TKI therapy (Moch et al. 2016; Vera-Badillo et al. 2015). In these studies, the objective response rate to targeted therapy was significantly lower in those with nccRCC, and both the PFS and OS times were shorter in patients with nccRCC as compared to ccRCC. These differences are probably due to different biological mechanisms of tumorigenesis and warrant further investigation (Vera-Badillo et al. 2015). Based on these studies, the panelists recommend sunitinib as first-line therapy for non-clear cell carcinomas (except for those of medullary and collecting duct histologies, which were not represented in the study) (recommendation level C) (Vera-Badillo et al. 2015).

Recently, important single-arm phase II trials evaluating immunotherapies in nccRCC were presented: (1) a study with atezolizumab plus bevacizumab (nccRCC and ccRCC with > 20% sarcomatoid differentiation, *N* = 65), with an ORR of 34% (McKay et al. 2019); (2) the Checkmate-427B cohort, which included 165 patients with nccRCC treated with single-agent pembrolizumab, with an ORR of 24.8% and a CR rate of 4.8% (Suárez et al. 2019); and (3) the Calypso trial, which evaluated the combination of savolitinib and durvalumab in papillary RCC, with an ORR of 27% and a PFS of 3.3 months (Powles et al. 2019a, b). Interestingly, the first two studies demonstrated a higher response rate in PDL1-positive tumors and a lower response rate in patients with chromophobe histology.

#### Papillary renal carcinoma

The most extensive data on treatment of papillary renal cancer (PRC) is in a phase II trial in which patients with nccRCC were randomly assigned to treatment with sunitinib or everolimus. Most of patients (66%) in that trial had papillary histology. Patients treated with sunitinib had a longer median PFS than those treated with everolimus (8.3 vs 5.6 months). However, in the same trial, patients with poor-risk disease exhibited better PFS outcomes with everolimus (Armstrong et al. 2015). Another phase II trial demonstrated that everolimus has activity in patients with papillary renal cancer, showing a PFS rate at 6 months of 34% (Escudier et al. 2016). The panelists recommend first-line sunitinib (recommendation level B) (Armstrong et al. 2015) and second-line everolimus (recommendation level B) (Armstrong et al. 2015; Escudier et al. 2016) for patients with mRCC who require systemic therapy. This approach is also endorsed by the RECORD-3 trial, in which everolimus was not noninferior to sunitinib as first-line therapy (Motzer et al. 2014). As the ASPEN trial (Armstrong et al. 2015) also included tumors with other histologies, the panelists recommend everolimus as second-line treatment except for tumors with sarcomatoid features and for medullary and collecting duct histologies (not represented in the trial) (recommendation level D) (Armstrong et al. 2015; Escudier et al. 2016; Motzer et al. 2014).

Bevacizumab, a humanized monoclonal antibody that binds VEGF, was tested in addition to erlotinib (an epidermal growth factor receptor inhibitor) in a phase II trial (Stamatakis et al. 2011) and updated at the 2014 European Organization for Research and Treatment of Cancer–National Cancer Institute–American Association for Cancer Research (EORTC-NCI-AACR) Symposium. The median PFS time was 7 months for patients with sporadic disease, and the ORR was approximately 30% among all patients (Srinivasan 2014).

There are also data on MET inhibitors in papillary renal carcinoma. Foretinib is a multitarget TKI that targets MET and VEGF receptors and was tested in a phase II trial, in which the median PFS time was 9 months and the ORR was 13.5%. The OS at 1 year was 70% (Choueiri et al. 2013). Savolitinib, another MET inhibitor, was evaluated in a phase II study and showed a partial response rate of 18% in patients with MET-driven papillary RCC, along with a median PFS time of 6 months (Choueiri et al. 2017), but the phase III trial comparing savolitinib with sunitinib in MET-driven papillary RCC recently stopped accrual because of issues related to the biomarker selection of the patients (Choueiri 2017). Recently, promising results of the phase II CALYPSO study, evaluating the combination of savolitinib and durvalumab—an anti-PDL1 inhibitor—in papillary RCC were presented (Powles et al. 2019a, b). It is important to emphasize that none of these therapies received regulatory approval; hence, the inclusion of patients in clinical trials should be considered the first option whenever possible. Currently, there are trials testing the role of immunotherapy and its combination with TKIs in this context (Powles et al. 2019a, b; Suárez et al. 2019).

#### Collecting duct (Bellini's duct) RCC

Collecting duct RCCs are usually treated similarly to urothelial carcinoma, because both histologies share histologic and genomic characteristics. In a phase II French study evaluating chemotherapy with gemcitabine plus cisplatin, tumor response was observed in 26% of the patients and the median OS was 10.5 months (Oudard et al. 2007). Therefore, the panelists recommend cytotoxic chemotherapy based on gemcitabine and cisplatin/carboplatin as first-line therapy for collecting duct RCC (recommendation level B) (Oudard et al. 2007; Zisman et al. 2002). Similar to patients with metastatic urothelial carcinoma, these patients may be treated with dose-dense MVAC and postplatinum anti-PD1 or anti-PDL1 agents, although the activity of these regimens, specifically for collecting duct carcinoma, is lacking (recommendation level D) (Oudard et al. 2007; Zisman et al. 2002).

#### Renal medullary carcinoma

Renal medullary carcinoma is a rare neoplasm (Coogan et al. 1998) and data on treatment are scarce. From single-center experience data, chemotherapy is usually administered in the first-line setting (Hakimi et al. 2007). Regimens based on platinum agents and gemcitabine have shown activity against this rare condition (Maroja Silvino et al. 2013; Walsh et al. 2010). Additionally, regimens based on anthracyclines have activity against this condition (Maroja Silvino et al. 2013). Therefore, the panelists recommend chemotherapy with gemcitabine and cisplatin/carboplatin as first-line therapy for renal medullary carcinoma (recommendation level B) (Coogan et al. 1998; Hakimi et al. 2007; Maroja Silvino et al. 2013; Walsh et al. 2010).

#### Renal cancer with sarcomatoid features

Published data suggest that the presence of sarcomatoid differentiation is an adverse prognostic factor for patients with mRCC regardless of the histologic subtype. In a retrospective cohort study, patients with a sarcomatoid component treated with an angiogenesis inhibitor exhibited an ORR of 19% (Golshayan et al. 2009). Notably, those responses were limited to patients with ccRCC and a sarcomatoid component of less than 20% (Golshayan et al. 2009).

sRCC have been shown to express PD-1/PD-L1 at a higher rate than RCCs without sarcomatoid features (Joseph et al. 2015). The literature also suggests that tumors presenting with a sarcomatoid component of greater than 20% have worse risk classifications (IMDC) and poorer prognoses (Kyriakopoulos et al. 2015). Therefore, the presence of sarcomatoid features in renal cancer patients has prognostic value (Joseph et al. 2015; Kyriakopoulos et al. 2015). The percentage of sarcomatoid features in the biopsy specimen has long been important in deciding on the type of regimen the oncologist should choose to treat this particular subtype of patients (Joseph et al. 2015; Kyriakopoulos et al. 2015).

However, more recent data from the IMmotion 150 (Atkins et al. 2017) and IMmotion 151 (Motzer et al. 2018a, b) trials suggests that the therapeutic benefit is maintained across the different stratification subgroup factors, such as sarcomatoid features. These data are in accordance with those of previous studies showing that sRCC arise from the dedifferentiation of carcinomatous renal cancers. In a preclinical study, sarcomatoid and carcinomatous tumors exhibited 42% somatic single-nucleotide variant (SSNV) concordance (Auvray et al. 2019).

When compared with carcinomatous tumors, sarcomatoid tumors show a higher SSNV burden, a higher oncogene frequency of nonsynonymous SSNVs and a higher frequency of the loss of heterozygosity (LOH) (Bi et al. 2016). The most interesting data showed that most SSNVs shared by sarcomatoid and carcinomatous tumors had already been identified in ccRCC genes, including SET domain containing 2 (SETD2), polybromo 1 (PBRM1), von Hippel–Lindau tumor suppressor (VHL) and phosphatase and tensin homolog (PTEN) (Bi et al. 2016).

Data from the CheckMate 214 trial suggest that patients with higher expression of PD-1/PD-L1 and intermediate- and poor-risk classification (IMDC), independent of the percentage of sarcomatoid features, respond better to immunotherapy (Atkins et al. 2017; Motzer et al. 2018a, b). A recent retrospective analysis of the same study showed a ORR of 56.7% in an intermediate-/poor-risk population with sarcomatoid features, which compared favorably to that of sunitinib (ORR 19.2%; *p* < 0.001) (McDermott et al. 2019). The median progression-free survival (PFS) time, according to investigator assessment, among intermediate- or poor-risk sarcomatoid patients treated with combined nivolumab and ipilimumab immunotherapy was 8.4 months, compared to 4.9 months for those treated with TKI therapy, with a hazard ratio of 0.61 (0.38–0.97) and a *p* value of 0.0329 (McDermott et al. 2019). The median OS time among intermediate- or poor-risk sarcomatoid patients and treated with immunotherapy was 31.2 months in the sunitinib group; HR (95% CI) 0.55; *p* < 0.0155) (McDermott et al. 2019).

A re-analysis divided the patients in the phase III JAVELIN Renal 101 trial according to sarcomatoid histology to assess the effect of avelumab plus axitinib versus sunitinib in this new cohort of patients with mRCC. Of the 886 patients included in the trial, 108 were positive for the sarcomatoid component. Of these, 47 patients received avelumab plus axitinib, and 61 received sunitinib. Patients treated with sunitinib had a slightly higher rate of positivity for PD-L1 than those treated with avelumab plus axitinib (85.2% vs 72.3%). Treatment with avelumab plus axitinib achieved a statistically significant improvement in the ORR compared to treatment with sunitinib (46.8% vs 21.3%; OR 3.2, 95% CI), and two patients with sRCC had a complete response with this therapy. In addition, the time to response and the duration of response were statistically improved with avelumab plus axitinib (1.6 and 2.4 months, respectively) (Lattanzi 2019). In different circumstances, the KEYNOTE-426 clinical trial revealed that approximately 18% of the patients studied had sarcomatoid characteristics. An analysis of this cohort showed that treatment with pembrolizumab plus axitinib had an ORR twice that of treatment with sunitinib alone (55.8% vs 29.5%). The OS and PFS rates were also improved (83.4% vs 79.5%—HR 0.58, 95% CI; and median not reached *vs* 8.4 months, respectively) (Zhu 2019).

As second-line treatment for patients with sarcomatoid features (after progression on first-line VEGF-TKIs, mTOR inhibitors or chemotherapy), the panelists recommend nivolumab according to data from the CheckMate 025 trial (recommendation level D) (Motzer et al. 2015a, b).

### Active surveillance in metastatic disease: when to choose it?

Most of the treatments described above for mRCC can generate at least an advantage in objective responses and/or extended PFS and/or OS in patients with metastatic disease. Although these regimens are the standard of care and improve the quality of life, they are not curative in the vast majority of patients (with rare exceptions for immunotherapies such as high-dose IL-2) (Fyfe et al. 1995). Furthermore, disease control implies chronic therapy, with successive lines of treatment administered over time. Therefore, at every treatment continuation or modification, the oncologist must weigh in the overall burden of treatment, including the toxicity, time commitment and costs, and/or best supportive care, including the psychological and physical implications.

It is known from clinical practice that there is a subset of patients with mRCC characterized by slow metastatic growth. This observation is reflected in the successful outcome of metastasectomy in these patients. Approximately 30% of patients who undergo metastasectomy for oligometastatic, slow-growing disease, are disease-free at 5 years (Dabestani et al. 2014). In one small prospective cohort study, treatment-naïve patients with mRCC were subjected to initial observation until disease progression and were then treated with the current standard-of-care treatment (Oliver et al. 1989). Interestingly, approximately 10% of the patients did not progress by the end of 12 months of active surveillance. In addition, the observation period did not negatively impact the treatment outcome. Subsequent interferon alpha therapy showed an ORR of 14%, which was identical to that of patients who started immediate treatment with interferon alpha (Oliver et al. 1989). These data suggest that there is a subpopulation of patients with mRCC that can safely undergo initial surveillance (Oliver et al. 1989).

In a systematic review of the literature, the role of metastasectomy was evaluated in 2350 patients who underwent this line of treatment (Dabestani et al. 2014). Interestingly, a correlation between good-risk disease, submission to metastasectomy and improved survival was identified in a few studies included in the systematic review, in an independent manner (Eggener et al. 2008; Staehler et al. 2010). More recent data have shown that a subset of patients with mRCC can be safely monitored before initiating systemic therapy. In this prospective phase II study, whose main objective was to characterize the time to start systemic therapy in patients with mRCC under active surveillance, 48 patients were followed for an average of 38.1 months. The average surveillance time from registration in the study to the beginning of systemic therapy was 14.9 months. During the study, 46% of patients died due to mRCC, and a shorter period of surveillance was associated with higher numbers of IMDC risk factors and higher numbers of metastatic disease sites (Rini et al. 2016). Therefore, the panelists recommend that asymptomatic, good-risk patients, and even intermediate-risk patients with asymptomatic, low-volume disease should be considered for active surveillance (recommendation level B) (Dabestani et al. 2014; Eggener et al. 2008; Hafez et al. 1997; Oliver et al. 1989; Staehler et al. 2010).

### Osteoclast inhibitors: which and when to indicate?

One of the main metastatic sites of RCC is bone. Bone metastasis has been observed in up to 31% of patients with mRCC (Seaman et al. 1996). Up to 91% of patients with bone metastasis present with localized pain secondary to lytic bone fractures (Loblaw et al. 2005).

In addition to bone pain, bone metastasis can add comorbidities that increase patient mortality rates and the number of interventional procedures (von Moos et al. 2013a, b). In a double-blind study, it was noted that approximately 8% of patients who needed hospital admission due to bone fractures or bone pain eventually died. The high mortality rate is thought to be due to complications of bone metastasis, such as the inability to regain the function of an impaired limb or infectious complications of surgical procedures in very ill patients (von Moos et al. 2013a, b). Therefore, the correct diagnosis, treatment and prevention of bone metastasis are of extreme importance in preserving the quality of life of cancer patients and preventing an increase in mortality rates due to cancer complications. To diagnose and study the various complications of bone metastasis, the clinician measures skeletal-related events (SREs), defined as the need for surgery or radiotherapy for pathologic fractures, spinal cord compression and malignant hypercalcemia (Wardley et al. 2005).

The management of patients with bone metastasis requires a multidisciplinary approach. Antineoplastic treatments, such as chemotherapy and endocrine therapy, surgery and radiotherapy are often needed to treat patients with bone metastasis. Supportive care therapies, such as osteoclast inhibitors, analgesics and electrolyte control agents, are essential adjuvants in this approach (Wardley et al. 2005).

There are two classes of osteoclast inhibitors that are often used in patients with bone metastases from solid tumors: bisphosphonates and denosumab. The use of these osteoclast inhibitors improves the quality of life (QoL), especially physical, emotional and social functioning (Henry et al. 2011). However, the choice between these two agents, which have different mechanisms of action, is the subject of literature studies. A few trials have compared denosumab with zoledronic acid in patients with various types of solid tumors except for breast and prostate cancers (Lipton et al. 2012).

A meta-analysis of phase III trials comparing zoledronic acid with denosumab in this setting concluded that denosumab was superior to zoledronic acid in reducing the risk of a first SRE by 17% and delaying the time to hypercalcemia of the malignancy (HR, 0.83; 95% CI, 0.76–0.90), although the OS and disease progression rates were not different between the treatments (Amadori et al. 2013). Another important characteristic of denosumab is the absence of renal toxicity, which is particularly important because a significant number of patients present with abnormal kidney function. Therefore, the panelists recommend denosumab as the agent of choice for the treatment of bone metastasis in patients with mRCC (recommendation level A) (Amadori et al. 2013; Lipton et al. 2012).

However, there are many patients who are already being treated with zoledronic acid due to its longstanding clinical availability. For those patients, the dosing frequency is also a theme in the literature. There are data from phase III placebo-controlled trials and noninferiority studies showing that 4 mg of zoledronic acid every 12 weeks is noninferior to 4 mg monthly in breast cancer patients (Himelstein et al. 2017; Hortobagyi et al. 2017; Sepulveda et al. 2002). The panelists could not reach consensus regarding the frequency of therapy with zoledronic acid, even though the 12-week regimen relates only to breast cancer patients with bone metastasis. After the second round of voting, 50% of the panelists recommended 4 mg monthly (recommendation level A) (Amadori et al. 2013; Himelstein et al. 2017), and the other half recommended 4 mg of zoledronic acid every 12 weeks (recommendation level B) (Amadori et al. 2013; Sepulveda et al. 2002).

### Exclusive best supportive care: when to adopt it?

Palliative care is a medical specialty that studies the best approaches for preventing and relieving suffering in patients and their families and for promoting the best possible quality of life (QOL) for individuals suffering from life-threatening illnesses (Wilson et al. 2007).

The source of suffering is broad (Cassell 1991). The biopsychosocial dimensions of disease and their burden are individually assessed and approached on a case-to-case basis, with the goal of relieving suffering and promoting an improved QoL (Cassell 1991). Therefore, the presence of pain or malignant recurrent hypercalcemia, the worsening of performance status and psychological suffering are important areas for the oncologist to assess in every patient possibly indicated for exclusive best supportive care (Cassell 1991; Richardson et al. 2007).

There are a vast number of instruments used to assess symptom burden, emotional distress and the necessity for palliative care (Buccheri et al. 1996). One of the most common instruments is the Eastern Cooperative Oncology Group Performance Scale (ECOG PS) (Oken et al. 1982), which uses a five-point scale based on the patient’s symptoms and is capable of accurately predicting prognosis. Based on these instruments, the panelists selected a combination that pushed the renal cell cancer patient towards exclusive supportive care. An ECOG-PS score of 3 or higher, untreatable malignant hypercalcemia, rapid clinical performance deterioration associated with a worsening ECOG-PS score and treatment with over 3 lines of renal cancer cell therapy are possible indications for exclusive supportive care (recommendation level A) (Buccheri et al. 1996; Cassell 1991; Oken et al. 1982; Richardson et al. 2007; Wilson et al. 2007).

## Final considerations

The understanding of renal cell cancer biology and treatment has increased exponentially in the last 2 decades, especially because of the clinical incorporation of targeted therapy and immunotherapy (Choueiri et al. 2015, 2018; Motzer et al. 2007, 2015a, b, 2018a; Powles et al. 2019a, b).

Medical research is of critical importance for advances in daily practice and improvements in clinical care worldwide. To achieve this goal, several medical societies and consensus groups regularly update their recommendations and make various efforts to spread new knowledge (Escudier et al. 2019; Graham et al. 2018).

This article presents the recommendations of the Latin American Cooperative Oncology Group–Genitourinary section (LACOG-GU) and Latin American Renal Cancer Group (LARCG) for renal cell cancer treatment. These recommendations were consistently based on the best possible clinical research evidence, preclinical data, or expert opinion to improve patient outcomes in an ever-changing oncologic landscape. Since the field is rapidly evolving, regular updates based on the results of ongoing clinical trials are needed in the future.

## Electronic supplementary material

Below is the link to the electronic supplementary material.

Supplementary file1 (DOCX 276 kb)
