# Supplementary material for: Advanced renal cell carcinoma (RCC) management: an expert panel recommendation from the Latin American Cooperative Oncology Group (LACOG) and the Latin American Renal Cancer Group (LARCG)
Source: J Cancer Res Clin Oncol. 2020 May 14;146(7):1829–45. doi: 10.1007/s00432-020-03236-4 (PMC7256074; doi:10.1007/s00432-020-03236-4)
Supplement: Supplementary file 1 — Supplementary file1 (DOCX 276 kb) [file 432_2020_3236_MOESM1_ESM.docx]

**SUPPLEMENTARY MATERIAL:** Results of the voting occurred in the expert meeting about the advanced renal cell carcinoma (RCC) management: first and second rounds

**Article Title:** Advanced renal cell carcinoma (RCC) management: an expert panel recommendation from the Latin American Cooperative Oncology Group (LACOG) and the Latin American Renal Cancer Group (LARCG)

**Journal Name:** Journal of Cancer Research and Clinical Oncology

**Author Names:** Andrey Soares^1,2,^*, Fernando Sabino Marques Monteiro^3,4^, Fernando Cotait Maluf^1,3,5^, Diogo Assed Bastos^6^, Denis Leonardo Jardim^6^, André Deeke Sasse^7^, Adriano Gonçalves e Silva^8^, André P. Fay^9,10^, Diogo Augusto Rodrigues da Rosa^11^, Evanius Wierman^12^, Fabio Kater^5^, Fabio A. Schutz^5^, Fernando Nunes Galvão de Oliveira^13^, Igor Alexandre Protzner Morbeck^14^, José Augusto Rinck Jr^15^, Karine Martins da Trindade^16,17^, Manuel Caitano Maia^18^, Vinicius Carrera Souza^19^, Deusdedit Cortez Vieira da Silva Neto^20^, Felipe de Almeida e Paula^21^, Fernando Korkes^1,22^, Gustavo Franco Carvalhal^9^, Lucas Nogueira^23^, Roni de Carvalho Fernandes^20,24^, Rodolfo Borges dos Reis^25^, Wagner Eduardo Matheus^26^, Wilson Francisco Schreiner Busato Jr^27^, Walter Henriques da Costa^15,24,28^, Stênio de Cássio Zequi^15,28^

**Affiliation:**

^1^Hospital Israelita Albert Einstein. Av. Albert Einstein, 627 - Morumbi, São Paulo/SP - CEP: 05652-900, Brazil

^2^Centro Paulista de Oncologia/Oncoclínicas. Av. Brigadeiro Faria Lima, 4300 - Vila Olímpia, São Paulo/SP - CEP: 01452-000, Brazil

^3^Hospital Santa Lúcia. SHLS 716 Conjunto C - Brasilia/DF - CEP: 70390-700, Brazil

^4^Hospital Universitário de Brasília. SGAN 605 - Brasilia/DF - CEP: 70840-901, Brazil

^5^Beneficência Portuguesa de São Paulo. R. Martiniano de Carvalho, 965 - Bela Vista, São Paulo/SP - CEP: 01323-001, Brazil

^6^Hospital Sírio-Libanês. R. Dona Adma Jafet, 91 - Bela Vista, São Paulo/SP - CEP: 01308-050, Brazil

^7^Grupo SOnHE. Av. Dr. Heitor Penteado, 1780 - Taquaral, Campinas/SP - CEP: 13075-460, Brazil

^8^Instituto do Câncer e Transplante de Curitiba (ICTR). R. Myltho Anselmo da Silva, 870 - Mercês, Curitiba/PR - CEP: 80510-130, Brazil

^9^Escola de Medicina e Hospital São Lucas da Pontifícia Universidade Católica do Rio Grande do Sul. Av. Ipiranga, 6690 - Prédio 60 - Partenon, Porto Alegre/RS - CEP: 90610-000, Brazil

^10^Grupo Oncoclínicas. R. Tobias da Silva, 126 - Moinhos do Vento, Porto Alegre/RS - CEP: 90570-020, Brazil

^11^Grupo Oncoclínicas. Praia de Botafogo, 300 - Botafogo, Rio de Janeiro/RJ - CEP: 22250-905, Brazil

^12^Instituto de Oncologia do Paraná. R. Mateus Leme, 2631/B - Centro Cívico, Curitiba/PR - CEP: 80520-174, Brazil

^13^CLION - GRUPO CAM. R. Altino Serbeto de Barros, 119 - Itaigara, Salvador/BA - CEP: 41810-570, Brazil

^14^Hospital Sírio-Libanês. SGAS 613, Centro Médico L2 - Asa Sul, Brasília/DF - CEP: 70200-730, Brazil

^15^AC Camargo Cancer Center. R. Professor Antônio Prudente, 211 - Liberdade, São Paulo/SP - CEP: 01509-010, Brazil

^16^Hospital São Carlos/Oncocentro. Av. Pontes Vieira, 2531 - Dionísio Torres, Fortaleza/CE - CEP: 60135-237, Brazil

^17^Santa Casa de Misericórdia de Fortaleza. R. Barão do Rio Branco, s/n - Centro, Fortaleza/CE - CEP: 60025-060, Brazil

^18^Centro de Oncologia do Paraná. Rodovia BR-277, 1437 - Ecoville, Curitiba/PR - CEP: 82305-100, Brazil

^19^Oncologia D'Or. Av. São Rafael, 2152, 6 Andar, Hospital São Rafael, São Marcos, Salvador/BA - CEP: 41253-190, Brazil

^20^Hospital Central da Santa Casa de Misericórdia de São Paulo. R. Dr. Cesário Mota Júnior, 112 - Vila Buarque, São Paulo/SP - CEP: 01221-020, Brazil

^21^Hospital Regional do Câncer de Presidente Prudente. Av. Coronel José Soares Marcondes, 2380 - Vila Euclides, Presidente Prudente/SP - CEP: 19013-050, Brazil

^22^ABC Medical School. Av. Príncipe de Gales, 821 - Príncipe de Gales, Santo André/SP - CEP: 09060-650, Brazil

^23^Hospital das Clínicas da Universidade Federal de Minas Gerais. Av. Prof. Alfredo Balena, 110 - Santa Efigência, Belo Horizonte/BH - CEP: 30130-100, Brazil

^24^Faculdade de Ciências Médicas da Santa Casa de São Paulo. R. Dr. Cesário Mota Jr., 61 - Vila Buarque, São Paulo/SP - CEP: 01221-020, Brazil

^25^Faculdade de Medicina de Ribeirão Preto - Universidade de São Paulo. Av. Bandeirantes, 3900 - Monte Alegre, Ribeirão Preto/SP - CEP: 14049-900, Brazil

^26^Faculdade de Ciências Médicas da Universidade Estadual de Campinas. R. Tessália Vieira de Camargo, 126 - Cidade Universitária Zeferino Vaz, Campinas/SP - CEP 13083-887, Brazil

^27^UNIVALI - Universidade do Vale do Itajaí. R. Uruguai, 458 - Centro, Itajaí/SC - CEP: 88302-901, Brazil

^28^National Institute for Science and Technology in Oncogenomics and Therapeutic Innovation, AC Camargo Cancer Center. R. Professor Antônio Prudente, 211 - Liberdade, São Paulo/SP - CEP: 01509-010, Brazil

***Correspondence:**

Andrey Soares, MD

Division of Oncology, Hospital Israelita Albert Einstein. Av. Albert Einstein, 627 - Morumbi, São Paulo/SP - CEP: 05652-900, Brazil

Division of Oncology, Centro Paulista de Oncologia/Oncoclínicas. Av. Brigadeiro Faria Lima, 4300 - Vila Olímpia, São Paulo/SP - CEP: 01452-000, Brazil

Phone: +55 (11) 98315-6449

E-mail: [dr.andrey@uol.com.br](mailto:dr.andrey@uol.com.br)

**1. Nephrectomy in Advanced/Metastatic Disease**

**1) Cytoreductive nephrectomy in all patients?**

1- Yes

2- No

0- Abstain

**2) In what group of patients: Low-risk only?**

1- Yes

2- No

0- Abstain

**3)** **In what group of patients: Low-risk?**

1- Yes

2- No

0- Abstain

**4) I recommend multidisciplinary discussion for cytoreductive nephrectomy decision making?**

1- Always

2- Never

3- In selected cases

0- Abstain

**5) Cytoreductive nephrectomy – intermediate- and good-risk patients?**

1- Yes

2- No

0- Abstain

**AFTER THE SECOND ROUND OF VOTING:**

**5) Cytoreductive nephrectomy – intermediate- and good-risk patients?**

1- Yes

2- No

0- Abstain

**6) Cytoreductive nephrectomy – intermediate-risk patients?**

1- Yes

2- No

3- In Selected Cases

0- Abstain

**7) Cytoreductive nephrectomy – poor-risk patients?**

1- Yes

2- No

3- In selected cases

0- Abstain

**AFTER THE SECOND ROUND OF VOTING:**

**7) Cytoreductive nephrectomy – poor-risk patients?**

1- Yes

2- No

3- In selected cases

0- Abstain

**8) I indicate cytoreductive nephrectomy for patients:**

1- Always before systemic treatment

2- Always after systemic treatment

3- Before systemic treatment only for patients with severe renal disease and without hepatic and/or bone metastases

0- Abstain

**AFTER THE SECOND ROUND OF VOTING:**

**8) I indicate cytoreductive nephrectomy for patients:**

1- Always before systemic treatment

2- Always after systemic treatment

3- Before systemic treatment only for patients with severe renal disease and without hepatic and/or bone metastases

0- Abstain

**9) If in the presence of tumor complications/bleeding and/or urinary obstruction and/or refractory pain and/or hypertensive crisis?**

1- Always

2- Never

3- Always under evaluation by the criteria and multidisciplinary discussion

0- Abstain

**10) Cytoreductive nephrectomy – clear cell and non-clear cell?**

1- Yes

2- No

3- Clear cell only

4- Non-clear cell only

0- Abstain

**After the second round of voting:**

**10) Cytoreductive nephrectomy – clear cell and non-clear cell?**

1- Yes

2- No

3- Clear cell only

4- Non-clear cell only

0- Abstain

**11) Cytoreductive nephrectomy – Central Nervous System Metastases?**

1- Yes

2- No

0- Abstain

**12) If cytoreductive nephrectomy is indicated in patients with brain metastases: for any risk group?**

1- Yes

2- No

0- Abstain

**13) Should the percentage of tumor volume to be removed compared to the metastatic volume be an indication criterion?**

1- Yes

2- No

0- Abstain

**2. Metastasectomy**

**14) What risk score will we adopt?**

1- IMDC

2- MOTZER/MSKCC

0- Abstain

**15) Should metastasectomy be performed when there is a single metastasis?**

1- Yes

2- No

0- Abstain

**16) Should metastasectomy be performed when there are multiple metastases?**

1- Yes

2- No

3- Depending on the location of the metastases

0- Abstain

**17) Should metastasectomy be performed for lymph node metastases?**

1- Yes

2- No

3- In most cases

4- In a minority of cases

0- Abstain

**AFTER THE SECOND ROUND OF VOTING:**

**17) Should metastasectomy be performed for lymph node metastases?**

1- Yes

2- No

3- In most cases

4- In a minority of cases

0- Abstain

**18) Should metastasectomy be performed for bone metastases?**

1- Yes

2- No

3- In most cases

4- In a minority of cases

0- Abstain

**AFTER THE SECOND ROUND OF VOTING:**

**18) Should metastasectomy be performed for bone metastases?**

1- Yes

2- No

3- In most cases

4- In a minority of cases

0- Abstain

**19) Should metastasectomy be performed when the disease-free interval is <1 year?**

1- Yes

2- No

3- In most cases

4- In a minority of cases

0- Abstain

**AFTER THE SECOND ROUND OF VOTING:**

**19) Should metastasectomy be performed when the disease-free interval is <1 year?**

1- Yes

2- No

3- In most cases

4- In a minority of cases

0- Abstain

**20)** **Should metastasectomy be performed when the disease-free interval is > 1 year?**

1- Yes

2- No

3- In most cases

4- In a minority of cases

0- Abstain

**AFTER THE SECOND ROUND OF VOTING:**

**20)** **Should metastasectomy be performed when the disease-free interval is > 1 year?**

1- Yes

2- No

3- In most cases

4- In a minority of cases

0- Abstain

**21)** **Should metastasectomy be performed in cases with local recurrence?**

1- Yes

2- No

3- In most cases

4- In a minority of cases

0- Abstain

**AFTER THE SECOND ROUND OF VOTING:**

**21)** **Should metastasectomy be performed in cases with local recurrence?**

1- Yes

2- No

3- In most cases

4- In a minority of cases

0- Abstain

**22) Metastasectomy synchronous to the primary site?**

1- Yes

2- No

3- In most cases

4- In a minority of cases

0- Abstain

**AFTER THE SECOND ROUND OF VOTING:**

**22) Metastasectomy synchronous to the primary site?**

1- Yes

2- No

3- In most cases

4- In a minority of cases

0- Abstain

**23) Should neoadjuvant treatment be performed?**

1- Yes

2- No

3- In most cases

4- In a minority of cases

0- Abstain

**24) In which IMDC risk group should metastasectomy be performed: good-risk?**

1- Yes

2- No

0- Abstain

**25)** **In which IMDC risk group should metastasectomy be performed: intermediate-risk?**

1- Yes

2- No

0- Abstain

**26)** **In which IMDC risk group should metastasectomy be performed: poor-risk?**

1- Yes

2- No

0- Abstain

**3. First-line treatment in metastatic disease**

**27) Is PD-L1 expression important for defining first-line systemic treatment of metastatic clear cell carcinoma?**

1- Yes

2- No

0- Abstain

**28) For intermediate risk disease?**

1- Nivolumab + Ipilimumab

2- Pembrolizumab + Axitinib

3- Nivolumab + Ipilimumab or Pembrolizumab + Axitinib

4- Sunitinib/Pazopanib

5- Bevacizumab + IFN

6- Cabozantinib

7- High doses of IL-2

0- Abstain

**29) For high risk disease?**

1- Nivolumab + Ipilimumab

2- Pembrolizumab + Axitinib

3- Nivolumab + Ipilimumab or Pembrolizumab + Axitinib

4- Sunitinib/Pazopanib

5- Bevacizumab + IFN

6- Cabozantinib

7- High doses of IL-2

8- Tensirolimus

0- Abstain

**AFTER THE SECOND ROUND OF VOTING:**

**29) For high risk disease?**

1- Nivolumab + Ipilimumab

2- Pembrolizumab + Axitinib

3- Nivolumab + Ipilimumab or Pembrolizumab + Axitinib

4- Sunitinib/Pazopanib

5- Bevacizumab + IFN

6- Cabozantinib

7- High doses of IL-2

8- Tensirolimus

0- Abstain

**30) For low risk disease?**

1. Nivolumab + Ipilimumab

2. Pembrolizumab + Axitinib

3. Sunitinib/Pazopanib

4. Bevacizumab + IFN

5. Cabozantinib

6. High doses of IL-2

7. No preference for “old” anti VEGF (sunitinib/pazopanib/bevacizumab + IFN), cabozantinib or pembrolizumab + axitinib

0- Abstain

**AFTER THE SECOND ROUND OF VOTING:**

**30) For low risk disease?**

1. Nivolumab + Ipilimumab

2. Pembrolizumab + Axitinib

3. Sunitinib/Pazopanib

4. Bevacizumab + IFN

5. Cabozantinib

6. High doses of IL-2

7. No preference for “old” anti VEGF (sunitinib/pazopanib/bevacizumab + IFN), cabozantinib or pembrolizumab + axitinib

0- Abstain

**31) I recommend cabozantinib as first-line treatment for intermediate- and high-risk patients with bone metastases as important sites of disease:**

1- Always

2- Never

3- In most cases

4- In a minority of cases

0- Abstain

**AFTER THE SECOND ROUND OF VOTING:**

**31) I recommend cabozantinib as first-line treatment for intermediate- and high-risk patient with bone metastases as important sites of disease:**

1- Always

2- Never

3- In most cases

4- In a minority of cases

0- Abstain

**32) Among antiangiogenic therapies, what is your preference?**

1- Sunitinib

2- Pazopanib

0- Abstain

**AFTER THE SECOND ROUND OF VOTING:**

**32) Among antiangiogenic therapies, what is your preference?**

1- Sunitinib

2- Pazopanib

0- Abstain

**33) The choice of antiangiogenic therapy as first line is due to the:**

1- My experience with the drug

2- Toxicity Profile

3- Cost

4- I believe is superior

5- Access

0- Abstain

**AFTER THE SECOND ROUND OF VOTING:**

**33) The choice of antiangiogenic therapy as first line is due to the:**

1- My experience with the drug

2- Toxicity Profile

3- Cost

4- I believe is superior

5- Access

0- Abstain

**34) With the arrival of combinations of antiangiogenic and immunotherapy (IO), or IO-IO therapy, I believe that high-dose IL2 therapy:**

1- No longer has a role in treatment

2- Has some role in selected cases

0- Abstain

**4. Second-line treatment in metastatic disease**

**35) For patients progressing after immunotherapy combo (Nivo + Ipi) in 1st line**

1- Sunitinib/Pazopanib

2- Axitinib

3- Everolimus

4- Cabozantinib

5- Lenvatinib + Everolimus

0- Abstain

**AFTER THE SECOND ROUND OF VOTING:**

**35) For patients progressing after immunotherapy combo (Nivo + Ipi) in 1st line**

1- Sunitinib/Pazopanib

2- Axitinib

3- Everolimus

4- Cabozantinib

5- Lenvatinib + Everolimus

0- Abstain

**36) For patients progressing after pembro and axitinib in first line:**

1- Sunitinib/Pazopanib

2- Axitinib

3- Everolimus

4- Cabozantinib

5- Lenvatinib + Everolimus

0- Abstain

**37) The choice of 2nd line therapy is due to:**

1- My experience with the drug

2- Toxicity Profile

3- Cost

4- I believe the drug is superior

5- Access

0- Abstain

**AFTER THE SECOND ROUND OF VOTING:**

**37) The choice of 2nd line therapy is due to:**

1- My experience with the drug

2- Toxicity Profile

3- Cost

4- I believe the drug is superior

5- Access

0- Abstain

**38) For patients who progress after initial treatment with a VEGF pathway inhibitor, I recommend:**

1- Sunitinib

2- Pazopanib

3- Sorafenib

4- Axitinib

5- Everolimus

6- Cabozantinib

7- Nivolumab

8- Lenvatinib + everolimus

0- Abstain

**39) I recommend nivolumab after VEGF inhibitor failure:**

1- Always

2- Never

3- In most cases

4- In a minority of cases

0- Abstain

**40)** **I recommend nivolumab after VEGF inhibitor failure due to:**

1- Experience with the drug

2- Cost

3- Profile and incidence of toxicity

4- Mechanism of action

5- I believe this drug has the best effectiveness data

6- Better control rate in the study

0- Abstain

**AFTER THE SECOND ROUND OF VOTING:**

**40)** **I recommend nivolumab after VEGF inhibitor failure due to:**

1- Experience with the drug

2- Cost

3- Profile and incidence of toxicity

4- Mechanism of action

5- I believe this drug has the best effectiveness data

6- Better control rate in the study

0- Abstain

**41) I recommend cabozantinib after anti-VEGF therapy failure:**

1- Always

2- Never

3- In most cases

4- In a minority of cases

0- Abstain

**42)** **I recommend cabozantinib after anti-VEGF therapy failure due to:**

1- Experience with the drug

2- Cost

3- Profile and incidence of toxicity

4- Mechanism of action

5- I believe this drug has the best effectiveness data

6- Better control rate in the study

0- Abstain

**43) I recommend a TKI (sunitinib/pazopanib) after anti-VEGF therapy failure:**

1- Always

2- Never

3- In most cases

4- In a minority of cases

0- Abstain

**AFTER THE SECOND ROUND OF VOTING:**

**43) I recommend a TKI (sunitinib/pazopanib) after anti-VEGF therapy failure:**

1- Always

2- Never

3- In most cases

4- In the minority of cases

0- Abstain

**44) I recommend a TKI (sunitinib/pazopanib) after anti-VEGF therapy failure due to:**

1- Experience with the drug

2- Cost

3- Profile and incidence of toxicity

4- Mechanism of action

5- I believe the drug has the best effectiveness data

6- Better control rate in the study

0- Abstain

**45) I recommend a TKI (axitinib) after anti-VEGF therapy failure:**

1- Always

2- Never

3- In most cases

4- In a minority of cases

0- Abstain

**AFTER THE SECOND ROUND OF VOTING:**

**45) I recommend a TKI (axitinib) after anti-VEGF therapy failure:**

1- Always

2- Never

3- In most cases

4- In a minority of cases

0- Abstain

**46)** **I recommend a TKI (axitinib) after anti-VEGF therapy failure due to:**

1- Experience with the drug

2- Cost

3- Profile and incidence of toxicity

4- Mechanism of action

5- I believe the drug has the best effectiveness data

6- Best control rate in the study

0- Abstain

**AFTER THE SECOND ROUND OF VOTING:**

**46)** **I recommend TKI (axitinib) after failure of VEGF therapy due to:**

1- Experience with the drug

2- Cost

3- Profile and toxicity’s incidence

4- Mechanism of action

5- I believe to be the best effectiveness data

6- Best control rate in the study

0- Abstain

**47) I recommend everolimus after anti-VEGF therapy failure:**

1- Always

2- Never

3- In most cases

4- In a minority of cases

0- Abstain

**48) I recommend lenvatinib + everolimus after anti-VEGF therapy failure:**

1- Always

2- Never

3- In most cases

4- In a minority of cases

0- Abstain

**AFTER THE SECOND ROUND OF VOTING:**

**48) I recommend lenvatinib + everolimus after anti-VEGF therapy failure:**

1- Always

2- Never

3- In most cases

4- In a minority of cases

0- Abstain

**49)** **I recommend lenvatinib + everolimus after anti-VEGF therapy failure due to:**

1- Experience with the drug

2- Cost

3- Profile and incidence of toxicity

4- Mechanism of action

5- I believe the drug has the best effectiveness data

6- Best control rate in the study

0- Abstain

**5. Third and further treatment lines for metastatic disease**

**50) For patients who have progressed after combined immunosuppressive treatment (IO + IO or IO + Axitinib) and a TKI in 2nd line, I recommend:**

1- TKI not previously used

2- mTOR inhibitor (everolimus)

3- Lenvatinib + Everolimus

0- Abstain

**51)** **For patients who progressed after treatment with cabozantinib (1st line) and Immunotherapy (Nivo in 2nd line):**

1- Sunitinib

2- Pazopanib

3- Sorafenib

4- Axitinib

5- Everolimus

6- Lenvatinib + Everolimus

0- Abstain

**AFTER THE SECOND ROUND OF VOTING:**

**51)** **For patients who progressed after treatment with cabozantinib (1st line) and Immunotherapy (Nivo in 2nd line):**

1- Sunitinib

2- Pazopanib

3- Sorafenib

4- Axitinib

5- Everolimus

6- Lenvatinib + Everolimus

0- Abstain

**52) For patients who progress after treatment with VEGF inhibitors and everolimus:**

1- Retreatment with a TKI (sunitinib/pazopanib)

2- Axitinib

3- Nivolumab

4- Cabozantinib

5- Lenvatinib + everolimus

6- Abstain

**6. First-line treatment for metastatic disease (non-clear cell histologies)**

***6.1 First-line treatment***

**53) Which treatment option is preferred (except for the sarcomatoid/collecting ducts)?**

1- Sunitinib

2- Pazopanib

3- Temsirolimus

4- Everolimus

0- Abstain

**54) Do you change the treatment if the percentage of sarcomatoid pattern is > 20%?**

1- Yes

2- No

0- Abstain

**55) Which treatment option is preferred for tumors with < 20% sarcomatoid pattern (low risk)?**

1- Sunitinib

2- Pazopanib

3- Sunitinib + gemcitabine

4- Temsirolimus

5- Everolimus

6- Atezolizumab + bevacizumab

7- Gemcitabine + doxorubicin

8- Nivolumab + ipilimumab

0- Abstain

**56) Which treatment option is preferred for tumors with > 20% sarcomatoid pattern?**

1- Sunitinib

2- Pazopanib

3- Sunitinib + gemcitabine

4- Temsirolimo

5- Everolimo

6- Atezolizumab + bevacizumab

7- Gemcitabine + doxorubicin

8- Nivolumab + ipilimumab

0- Abstain

**AFTER THE SECOND ROUND OF VOTING:**

**56) Which treatment option is preferred for tumors with > 20% sarcomatoid pattern?**

1- Sunitinib

2- Pazopanib

3- Sunitinib + gemcitabine

4- Temsirolimus

5- Everolimus

6- Atezolizumab + bevacizumab

7- Gemcitabine + doxorubicin

8- Nivolumab + ipilimumab

0- Abstain

**57) Which treatment option is preferred for tumors of the collecting ducts (Bellini’s duct)?**

1- Sunitinib

2- Pazopanib

3- Temsirolimus/everolimus

4- Gemcitabine + cisplatin or carboplatin

5- mVAC

6- ddMVAC

0- Abstain

**58) Which treatment option is preferred for medullary renal tumors?**

1- Sunitinib

2- Pazopanib

3- Temsirolimus

4- Everolimus

5- Gemcitabine + cisplatin or carboplatin

6- mVAC

7- ddMVAC

0- Abstain

**59) Which treatment option is preferred for papillary tumors (type I and II)?**

1- Sunitinib

2- Pazopanib

3- Temsirolimus

4- Everolimus

5- Erlotinib + bevacizumab

0- Abstain

***6.2 Second-line treatment***

**60) Which treatment option is preferred after TKI (except for sarcomatoid pattern and collecting duct tumors)?**

1- Temsirolimus

2- Everolimus

3- Nivolumab

4- Cabozantinib

0- Abstain

**61) Which treatment option is preferred after mTOR inhibition (except for sarcomatoid, medullary, and collecting duct tumors)?**

1- Sunitinib

2- Pazopanib

3- Nivolumab

4- Cabozantinib

0- Abstain

**62) Which treatment option is preferred after TKI or TKI + chemotherapy in sarcomatoid pattern tumors?**

1- Axitinib

2- Temsirolimus

3- Everolimus

4- Nivolumab

0- Abstain

**63) Which treatment option is preferred after mTOR inhibitor therapy in sarcomatoid pattern tumors?**

1- Sunitinib

2- Pazopanib

3- Nivolumab

0- Abstain

**AFTER THE SECOND ROUND OF VOTING:**

**63) Which treatment option is preferred after mTOR inhibitor therapy in sarcomatoid pattern tumors?**

1- Sunitinib

2- Pazopanib

3- Nivolumab

0- Abstain

**64) Which treatment option is preferred after chemotherapy in sarcomatoid pattern tumors?**

1- Sunitinib

2- Pazopanib

3- Temsirolimus

4- Everolimus

5- Nivolumab

0- Abstain

**AFTER THE SECOND ROUND OF VOTING:**

**64) Which treatment option is preferred after chemotherapy in sarcomatoid pattern tumors?**

1- Sunitinib

2- Pazopanib

3- Temsirolimus

4- Everolimus

5- Nivolumab

0- Abstain

**65) Which treatment option is preferred after immunotherapy + anti-VEGF therapy in sarcomatoid pattern tumors?**

1- Sunitinib

2- Pazopanib

3- Axitinib

4- Temsirolimus

5- Everolimus

0- Abstain

**AFTER THE SECOND ROUND OF VOTING:**

**65) Which treatment option is preferred after immunotherapy + anti-VEGF therapy in sarcomatoid pattern tumors?**

1- Sunitinib

2- Pazopanib

3- Axitinib

4- Temsirolimus

5- Everolimus

0- Abstain

**66) Which treatment option is preferred for papillary tumors (type I and II) after VEGF inhibitor failure?**

1- Temsirolimus

2- Everolimus

3- Erlotinib + bevacizumab

0- Abstain

**AFTER THE SECOND ROUND OF VOTING:**

**66) Which treatment option is preferred for papillary tumors (type I and II) after VEGF inhibitor failure?**

1- Temsirolimus

2- Everolimus

3- Erlotinib + bevacizumab

0- Abstain

**67) What treatment option is preferred for papillary tumors (type I and II) after mTOR inhibitor failure?**

1- Sunitinib

2- Pazopanib

3- Erlotinib + bevacizumab

0- Abstain

**68) What treatment option is preferred for papillary tumors (type I and II) after erlotinib + bevacizumab failure?**

1- Sunitinib

2- Pazopanib

3- Temsirolimus

4- Everolimus

0- Abstain

**7. Active surveillance in metastatic disease: when to indicate?**

**69) Watchful waiting in metastatic disease: when to indicate?**

1- Only in good-risk, asymptomatic patients

2- Only after resection of all metastatic lesions, independent of risk

3- In asymptomatic, good-risk patients after resection of all metastatic lesions

4- Never

0- Abstain

**AFTER THE SECOND ROUND OF VOTING:**

**69) Watchful waiting in metastatic disease: when to indicate?**

1- Only in good-risk, asymptomatic patients

2- In good-risk, asymptomatic patients and in selected patients with asymptomatic, intermediate-risk, low-volume disease

3- Only after resection of all metastatic lesions, independent of risk

4- In asymptomatic, good-risk patients after resection of all metastatic lesions

0- Abstain

**8. Osteoclast inhibitors: which and when to indicate?**

**70) Metastatic bone disease – preferred agent?**

1- Zoledronic acid

2- Denosumab

3- If there is no contraindication to any agent, I have no preference

0- Abstain

**AFTER THE SECOND ROUND OF VOTING:**

**70) Metastatic bone disease – preferred agent?**

1- Zoledronic acid

2- Denosumab

3- If there is no contra-indication to any agent, I have no preference

0- Abstain

**71) Metastatic bone disease – Denosumab 120 mg, IV?**

1- Monthly

2- Never

0- Abstain

**72) Metastatic bone disease – Zoledronic acid, 4 mg, IV?**

1- Every 12 weeks

2- Monthly

3- Never

0- Abstain

**AFTER THE SECOND ROUND OF VOTING:**

**72) Metastatic bone disease – Zoledronic acid, 4 mg, IV?**

1- Every 12 weeks

2- Monthly

3- Never

0- Abstain

**9. Exclusive Best Supportive Care: when to adopt it?**

**73) Exclusive best supportive care – when do I indicate it?**

1- ECOG-PS 2 or higher

2- ECOG-PS 3 or higher

3- For untreatable malignant hypercalcemia

4- After second-line therapy (VEGFi and/or immunotherapy)

5- After 3 or more lines of therapy

6- For patients with fast symptomatic clinical deterioration, independent of the number of treatment lines

7- All of the above

8- None of the above

0- Abstain

**AFTER THE SECOND ROUND OF VOTING:**

**73) Exclusive best supportive care – when do I indicate it?**

1- PS-ECOG 3 or higher

2- For untreatable malignant hypercalcemia

3- After 3 or more lines of therapy

4- For patients with fast symptomatic clinical deterioration and loss of performance

5- All of the above

6- None of the above

0- Abstain
